# Management of financial conflicts of interests in clinical practice guidelines in Germany: results from the public database *GuidelineWatch*

**DOI:** 10.1186/s12910-018-0309-y

**Published:** 2018-06-28

**Authors:** Hendrik Napierala, Luise Schäfer, Gisela Schott, Niklas Schurig, Thomas Lempert

**Affiliations:** 1 Charité Universitätsmedizin, Berlin, Germany; 2 Drug Commission of the German Medical Association, Berlin, Germany; 3 Family Practice, Rastatt, Germany; 4 Department of Neurology, Schlosspark-Klinik, Berlin, Germany

**Keywords:** Clinical practice guidelines, Conflict of interest, Transparency, Regulation

## Abstract

**Background:**

The reliability of clinical practice guidelines has been disputed because guideline panel members are often burdened with financial conflicts of interest (COI). Current recommendations for COI regulation advise not only detailed declaration but also active management of conflicts. To continuously assess COI declaration and management in German guidelines we established the public database *LeitlinienWatch (GuidelineWatch)*.

**Methods:**

We analyzed all German guidelines at the highest methodological level (S3) that included recommendations for pharmacological therapy (*n* = 67) according to five criteria: declaration and assessment of COI, composition of the guideline development group, independence of the coordinators and lead authors, imposed abstentions because of COI and public external review. Each criterion was assessed using predefined outcome categories.

**Results:**

Most guidelines (76%) contained a detailed declaration of COI. However, none of the guidelines provided full transparency of COI assessment results. The guideline group was composed of a majority of participants with COI in 55% of the guidelines, no guideline was free of participants with COI. Only 9% of guidelines had coordinators and lead authors without any financial COI. Most guidelines (70%) did not provide a rule for abstentions for participants with COI. In 21% of guidelines there was a rule, but abstentions were either not practiced or not documented, whereas in 7% partial abstentions and in 2% complete abstentions were documented. Two thirds of the guideline drafts (67%) were not externally reviewed via a public website.

**Conclusions:**

COI are usually documented in detail in German guidelines of the highest methodological level. However, considerable improvement is needed regarding active management of COI, including recruitment of independent experts for guideline projects, abstention from voting for participants with COI and external review of the guideline draft. We assume that the publicly available ratings on *GuidelineWatch* will improve the handling of conflicts of interest in guideline development.

**Electronic supplementary material:**

The online version of this article (10.1186/s12910-018-0309-y) contains supplementary material, which is available to authorized users.

## Background

Conflicts of interest (COI) are ubiquitous and may threaten the integrity of medicine when not appropriately managed [1]. They may arise from professional interests, from personal or institutional relations and from financial ties to drug and device manufacturers. Financial COI deserve special attention because they are driven by a powerful industry which systematically seeks to influence medical evidence production, publication and dissemination for its advantage [2]. Establishing financial relations with key opinion leaders as advisors, speakers and, ultimately, guideline authors is one of the most effective strategies to achieve this goal. Numerous studies have shown that financial ties to industry are a risk factor for tainted views and practices in medicine [3–5].

Clinical practice guidelines are an essential tool to translate evidence-based medicine into practice. By the same token, they constitute an attractive target for industry intervention because a single guideline recommendation for or against a drug may determine its economic success or failure [6]. Therefore, guideline developers need to implement management strategies to protect guidelines from undue influence. The international debate on COI has centered on five principles to manage COI in guideline projects: detailed declaration of COI with independent evaluation, recruiting of panelists without COI, independent chairpersons, abstention from voting for participants with COI and public review of the guideline draft [7–10]. However, analysis of published guidelines reveals that COI are pervasive and rarely actively managed [11–14]. Two studies from Germany showed adequate declaration of COI in most guidelines but a lack of appropriate measures to minimize their influence on guideline content [15, 16].

This unsatisfactory situation prompted us to establish the website *GuidelineWatch* (in German: *LeitlinienWatch*) in December 2015 [17]. Our aim was to assess COI management of German guidelines according to the five principles mentioned above. Beyond previous analyses, our project provides public visibility of individual guidelines regarding their COI regulations, feedback for the authors and medical societies as well as best practice examples which may all serve to promote improved COI management. In the following, we report COI management of all 67 German guidelines at the methodologically highest level (S3) relating to pharmaceutical products.

## Methods

### The GuidelineWatch project

The website *GuidelineWatch* [17] was launched in December 2015 by three German organizations related to the No Free Lunch and anticorruption movement: MEZIS, NeurologyFirst, and Transparency International Germany. The primary aim of *GuidelineWatch* is to provide data on the quality of COI regulation in individual German guidelines and to make this information publicly accessible. All assessments are based on the official guideline documents including declarations of interests, method reports, and the guidelines as published by the Association of Scientific Medical Societies in Germany (Arbeitsgemeinschaft der Wissenschaftlichen Medizinischen Fachgesellschaften, AWMF). When an evaluation of a guideline is published on *GuidelineWatch* the medical society which produced the guideline is informed and offered the opportunity to comment on the website if they disagree with the evaluation. Factual errors are corrected by *GuidelineWatch*. On the website, results are presented as scores for each criterion and a summary score. For this paper we have restricted the analysis to descriptive categories.

### Guideline inclusion

The current report focuses on S3 guidelines, the highest methodological level in the AWMF system which requires both a broad professional and patient representation in the guideline panel and a systematic approach to evidence extraction. We included all S3-guidelines displayed in the AWMF register (www.awmf.org) on the 1st of January 2016 (*n* = 138). We excluded all expired guidelines (*n* = 29) and those which did not focus on recommendations for pharmacological therapy (*n* = 42). Thus, 67 guidelines were selected for this analysis.

### Assessment criteria

The evaluations of *GuidelineWatch* are based on five criteria: Declaration and assessment of COI, composition of the guideline development group, independence of the coordinators and lead authors, imposed abstentions because of COI and external review of the guideline draft. These criteria were selected as they reflect current national and international recommendations on COI management in guidelines [7, 9, 10] and because they can be readily assessed from the published guideline documents. Independent financing of the guideline was not added as a criterion because industry funding is an exclusion criterion for the AWMF register. The AWMF declaration of financial interests includes the following relationships: advisory boards, speaker honoraria, research funding, ownership or stocks of a healthcare company and personal relations to an authorized representative of the healthcare industry [18]. The five criteria and the predefined outcome categories are listed in Table 1. Outcomes are ranked from poor to good (A to E).

**Table 1:** Criteria and outcome categories of GuidelineWatch

Criterion	Outcome	
1. Declaration and assessment of COI	A	No declaration statement
	B	Undetailed declaration (yes/no)
	C	Detailed declaration stating companies
	D	Detailed declaration and external evaluation of COI (as opposed to self-assessment of the relevance of COI)
2. Composition of the guideline group	A	Lack of declarations
	B	More than 50% with financial COI
	C	25 to 50% with COI
	D	Less than 25% with COI
	E	All participants with full declarations and without COI
3. Independence of the coordinators and lead authors	A	Lack of declarations
	B	All coordinators/lead authors with COI
	C	Some of the coordinators/lead authors with COI
	D	All coordinators/lead authors with full declarations and without COI
4. Abstentions in participants with COI	A	No rule for abstentions
	B	Rule for abstentions, but no factual abstentions or voting results not documented
	C	Partial abstentions
	D	Fully documented abstentions of participants with COI
5. External public review	A	No external review
	B	External review on a publicly accessible website, not fully documented
	C	External review and documented handling of the individual comments

### Assessment procedure

Two primary reviewers analyzed each guideline. All assessments were finally reviewed by the senior author of this study (T.L.) for completeness and correct referencing to the original guideline documents. The review team included 10 physicians and two medical students with prior expertise in COI management. Reviews on *GuidelineWatch* are anonymous. A list of reviewers with affiliations and conflicts of interests is accessible on the website *GuidelineWatch/LeitlinienWatch *[17]. Reviewers are excluded from analyzing guidelines when they have financial COI relating to products that are relevant for the guideline. For each evaluation, only one primary reviewer was allowed to be a member of a medical society involved in the guideline project. None of the reviewers had personally participated in the development of a guideline analyzed by *GuidelineWatch*.

The two primary reviewers performed reviews independently. To assess COI management, they used only information available from the published guideline documents. Disagreement was resolved by discussion between reviewers and by the final reviewer (T.L.) in equivocal situations.

## Results

**Fig. 1:**
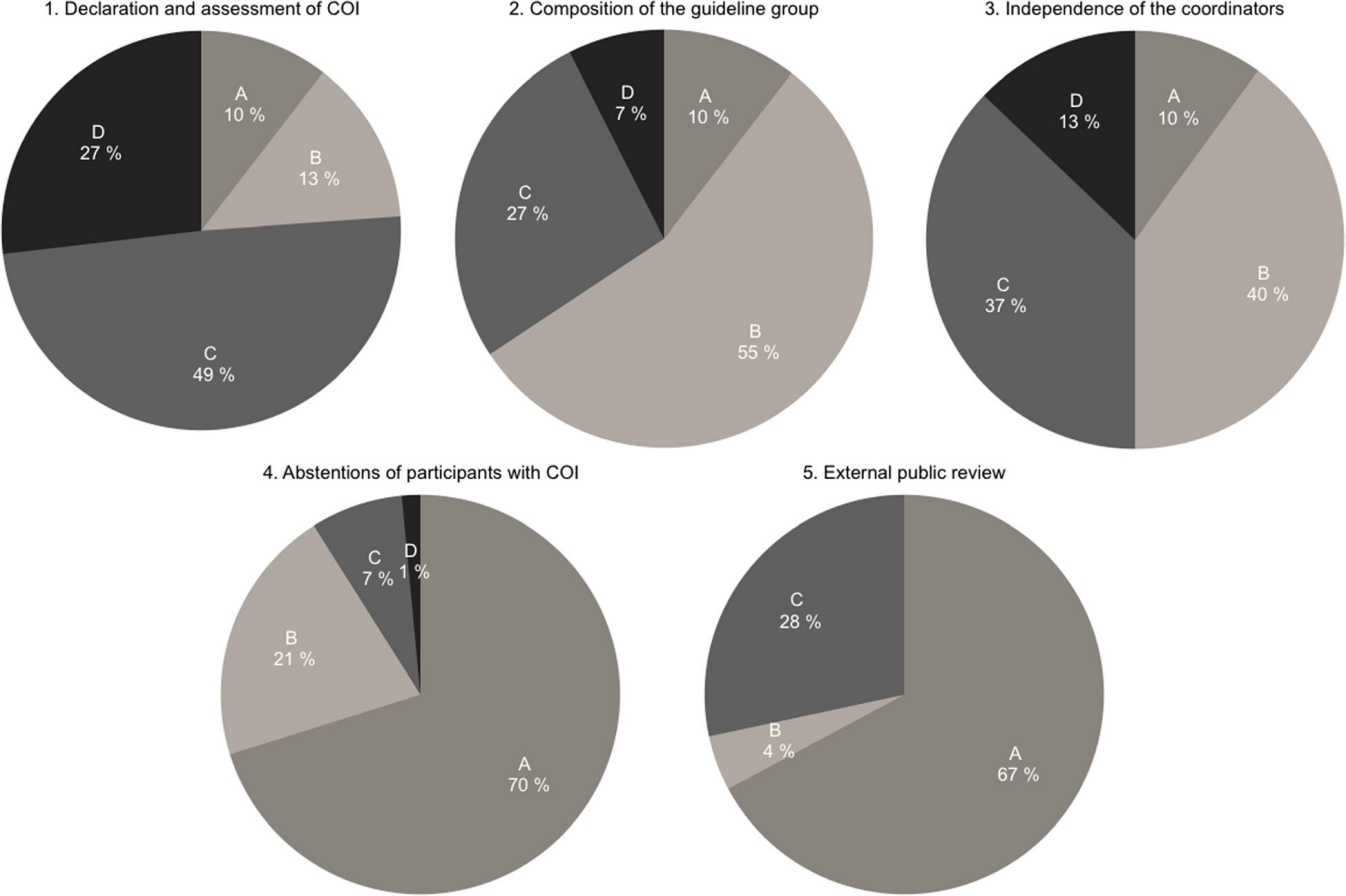
Conflicts of interests in 67 German guidelines: declaration and management Outcome categories are ranked from A (worst) to C or D (best) Declaration and Assessment: A. No declaration, B. Undetailed declaration, C. Detailed declaration, D. Detailed declaration plus independent assessment Composition of guideline group: A. Lack of declarations, B. More than 50% with COI, C. 25–50% with COI, D: Less than 25% with COI, E. All participants without COI (not depicted because the criterion was not met) Independence of coordinators: A. Lack of declarations, B. All coordinators with COI, B. Some coordinators with COI, C. All coordinators without COI Abstentions in participants with COI: A. No rule for abstentions, B. Rule for abstentions but not practiced, C. Partial abstentions D. Complete abstentions of participants with COI External public review: A. No external review, B. External review, not fully documented, C. External review, fully documented

We identified 138 S3-guidelines in the AWMF register. After excluding 29 expired guidelines and 42 guidelines not containing recommendations for pharmacotherapy we evaluated 67 guidelines published between 2011 and 2015 (Fig. 1; Additional file 1). Between two and 54 organizations (median: 13) were involved in the development of each guideline, most of them being medical societies but also patient advocacy groups and other medical organizations including the German Agency for Quality in Medicine and the Drug Commission of the German Medical Association.

### Declaration and assessment of COI

Most guidelines (76%; *n* = 51/67) contained a detailed declaration of COI stating companies and the type of financial relationship. These conflicts were self-assessed by the participants in 49% (*n* = 33/67) of the guidelines while the other 27% (18/67) had an external evaluation of COI. Undetailed declarations providing only yes/no answers for the various relationships were found in 14% (9/67) while 10% (7/67) of the guidelines did not contain any COI declaration.

### Composition of the guideline group

As COI declarations were lacking in 10% (7/67) of guidelines (see above) the prevalence of COI could not be assessed in these guideline groups. A majority of participants with COI was observed in 55% (37/67) of the guidelines. Twenty seven percent (18/67) of the guidelines had a proportion of 25–50% of participants with COI, while 8% (5/67) had a proportion of less than 25% with COI. No guideline was free of participants with COI.

### Independence of the coordinators and lead authors

The prevalence of COI in coordinators and lead authors could not be estimated in 10% (7/67) of the guidelines as detailed information on COI was not provided in the published documents. In 42% (28/67) all coordinators and lead authors had COI, in 39% (26/67) some had conflicts and only 9% (6/67) had coordinators and lead authors without any financial COI.

### Abstentions from voting in participants with COI

The majority of guidelines (70%; 47/67) did not provide a rule for abstentions for participants with COI. In 21% (14/67) there was a rule, but abstentions were either not practiced or not documented. Seven percent (5/67) documented partial abstentions while 2% (1/67) documented complete abstentions from voting for certain recommendations when there was COI. However, none of the guidelines provided full transparency of COI assessment results detailing not only companies but also drugs which were considered relevant and proposing consequences for affected members of the group. Thus, the (few) practiced abstentions were not backed by full information on who was excluded and why.

### External public review

Two thirds of the guideline drafts (67%; 45/67) were not externally reviewed via a public website. Five percent (3/67) described a review process but did not provide results while 28% (19/67) had an external review, documented the comments and described how each comment was handled.

## Discussion

### Main findings

With* GuidelineWatch* a publicly accessible website was established which continuously monitors the handling of conflicts of interest in German guidelines. Our analysis of COI management in 67 German top-level guidelines identified comprehensive disclosure of COI for most guidelines but a lack of active measures to reduce the impact of COI on guideline recommendations. Specifically, few guidelines documented results from COI assessments, excluded conflicted individuals from discussions or from voting or provided a public external review of draft guidelines.

### Strengths and weaknesses

To our knowledge *GuidelineWatch* is the first website to openly monitor the handling of conflicts of interest in guideline development. We regularly inform the medical societies about our evaluation and provide information on how to improve the handling of conflicts of interest.

A limitation of our study is the uncertainty regarding the true prevalence of COI. In this study we relied on the declarations of COI as published by the AWMF and did not research COI through external sources. However, incomplete disclosure of relevant financial relationships is common and may lead to an underestimation of COI [12, 19]. In contrast, COI may be overestimated when declared interests are not independently assessed for their relevance to guideline content which applied to more than two thirds of the 67 guidelines.

Another shortcoming is the restriction of our analysis to individual COI neglecting the dependency of many medical societies on industry funding. Financial relationships with biomedical companies were found in 63% of medical societies producing guidelines [20]. They are rarely disclosed in the guideline documents but may influence recommendations [20, 21]. One should also remember that bias in favor of industrial sponsors infiltrates guidelines not only by COI of the authors but also via distorted evidence production and dissemination [2].

### Context of other work

Previous studies mostly focused on prevalence and declaration of COI. High rates of guideline authors with COI seem to be a universal finding [11–14] while open declaration of COI varies between countries from 2% of guidelines in Denmark [22] to 93% in Germany [16].

Isolated declaration of COI without adequate management has been criticized as useless or even counterproductive with regard to bias reduction [23, 24]. One problem is the poor “signal-to-noise-ratio” of COI disclosures, i.e. hiding the relevant COI in a flood of declared financial relationships. Others are psychological in nature, e.g. authors perceiving COI declaration as a license for disseminating biased views or readers trusting authors despite declared COI because they do not expect to be misled [24].

Reduction of bias from COI starts with a serious effort to recruit independent authors as recommended by the Guidelines International Network [9]. It is worth noting that none of the analyzed guideline documents declared that such an effort had been made. Consequently, about half of the guideline groups and the coordinating teams had a majority of participants with COI. The Institute of Medicine in the U.S. proposed that medical societies should encourage their high-profile members to divest from industry relationships to create a sufficient reservoir of qualified and independent authors [7]. This strategy was recently put into practice by the Drug Commission of the German Medical Association.

COI need to be assessed by an independent committee which was performed in less than a third of evaluated German S3-guidelines. COI assessment is both in the interest of guideline authors and users as it separates the wheat from the chaff and guides further measures to reduce the impact of COI on specific recommendations. Results of COI assessments should be documented in the guideline highlighting companies and products relevant to the guideline content. This should be complemented by individualized decisions taken by the committee on how to deal with these conflicts. None of the analyzed guidelines provided such a detailed assessment.

Active COI management includes various measures ranging from complete exclusion from the guideline process, exclusion from certain discussions and abstention from voting on recommendations related to the COI [7, 9, 10]. Our study identified only few guidelines practicing abstentions which exposes the prevalent failure of pro-active COI management. An unresolved issue is the grading of COI as a prerequisite to guide appropriate measures [9, 10]. For example, participation in an advisory board may be regarded as a severe COI because advisory contracts commit the expert to the interest of the company. This would preclude the affected individual from participating in the guideline process. More often, however, advisory contracts are rated as moderate or even as mild COI which would require only abstention from voting on recommendations related to the conflict - or have no consequences at all.

A review of the guideline draft via a public website was performed by only one third of the guideline projects, which testifies to the underuse of an appropriate tool to counterbalance COI. By documenting how they judged and handled each comment guideline makers can appreciate and encourage external contributions, thus transforming a guideline draft into a project of the larger scientific community.

### Policy implications

We believe that a public website that continuously evaluates the handling of conflicts of interest in guideline development may promote active COI management as opposed to conventional COI declaration without consequence. Display of our results on the *GuidelineWatch* website, press and TV coverage as well as direct communication with guideline coordinators and medical societies has contributed to put COI management of guidelines on the agenda in Germany. In 2018, the German Association of Scientific Medical Societies has published reformed rules [10] which take up recommendations of the Institute of Medicine and the Guidelines International Network [7, 9]. In fact, several AWMF-guidelines published in 2018 show a trend towards improved COI management including detailed COI assessments and practiced abstentions. We hope that our ongoing public evaluation of COI management will motivate guideline groups to comply with the new regulations.

## Conclusion

*GuidelineWatch* creates a public platform in which the handling of conflicts of interest in German guidelines is continuously documented and monitored to improve the integrity, trustworthiness und credibility of clinical practice guidelines.

## Additional file

Additional file 1: Conflict of interest management in 67 German guidelines. This file lists the titles of all analysed guidelines and the detailed results for each guideline according to the five assessment criteria. (XLSX 18 kb)

## Abbreviations

AWMF: Arbeitsgemeinschaft der Wissenschaftlichen Medizinischen Fachgesellschaften, Association of Scientific Medical Societies in Germany
COI: Conflict(s) of interest
